# *catena*-Poly[[bis­[3-(1*H*-imidazol-1-yl)-1-phenyl­propan-1-one-κ*N* ^3^]nickel(II)]-μ-oxalato-κ^4^ *O* ^1^,*O* ^2^:*O* ^1′^,*O* ^2′^]

**DOI:** 10.1107/S1600536811049646

**Published:** 2011-11-25

**Authors:** Jian-Hua Guo

**Affiliations:** a Tianjin Key Laboratory of Structure and Performance for Functional Molecules, College of Chemistry, Tianjin Normal University, Tianjin 300387, People’s Republic of China

## Abstract

In the title compound, [Ni(C_2_O_4_)(C_12_H_12_N_2_O)_2_]_*n*_, the Ni^II^ atom, lying on a twofold rotation axis, is coordinated by two N atoms from two monodentate 3-(1*H*-imidazol-1-yl)-1-phenyl­propan-1-one (*L*) ligands and four O atoms from two oxalate anions in a distorted octa­hedral geometry. The oxalate anion has a twofold rotation axis through the mid-point of the C—C bond and acts as a bridging ligand, linking the Ni^II^ atoms into a polymeric chain along [010]. Weak inter­molecular C—H⋯O hydrogen bonds connect the chains, resulting in a three-dimensional supra­molecular structure. >

## Related literature

For background to the construction of metal-organic frameworks using a mixed-ligand strategy, see: Du *et al.* (2005 ▶); Tao *et al.* (2000 ▶); Ye *et al.* (2005 ▶).
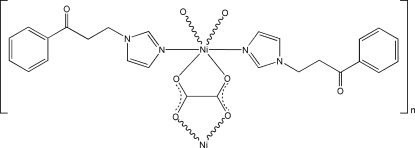


## Experimental

### 

#### Crystal data

[Ni(C_2_O_4_)(C_12_H_12_N_2_O)_2_]
*M* *_r_* = 547.20
Monoclinic, 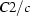

*a* = 15.3065 (11) Å
*b* = 5.6605 (4) Å
*c* = 27.536 (2) Å
β = 95.613 (1)°
*V* = 2374.3 (3) Å^3^
*Z* = 4
Mo *K*α radiation
μ = 0.87 mm^−1^
*T* = 296 K
0.28 × 0.22 × 0.20 mm

#### Data collection

Bruker APEXII CCD diffractometer
Absorption correction: multi-scan (*SADABS*; Sheldrick, 1996 ▶) *T*_min_ = 0.793, *T*_max_ = 0.845
5794 measured reflections
2105 independent reflections
1676 reflections with *I* > 2σ(*I*)
*R*_int_ = 0.043

#### Refinement

*R*[*F* ^2^ > 2σ(*F* ^2^)] = 0.045
*wR*(*F* ^2^) = 0.096
*S* = 1.02
2105 reflections
168 parameters
1 restraint
H-atom parameters constrained
Δρ_max_ = 0.63 e Å^−3^
Δρ_min_ = −0.45 e Å^−3^

### 

Data collection: *APEX2* (Bruker, 2007 ▶); cell refinement: *SAINT* (Bruker, 2007 ▶); data reduction: *SAINT*; program(s) used to solve structure: *SHELXS97* (Sheldrick, 2008 ▶); program(s) used to refine structure: *SHELXL97* (Sheldrick, 2008 ▶); molecular graphics: *XP* in *SHELXTL* (Sheldrick, 2008 ▶) and *DIAMOND* (Brandenburg & Berndt, 1999 ▶); software used to prepare material for publication: *SHELXTL*.

## Supplementary Material

Crystal structure: contains datablock(s) global, I. DOI: 10.1107/S1600536811049646/hy2488sup1.cif

Structure factors: contains datablock(s) I. DOI: 10.1107/S1600536811049646/hy2488Isup2.hkl

Additional supplementary materials:  crystallographic information; 3D view; checkCIF report

## Figures and Tables

**Table 1:** Hydrogen-bond geometry (Å, °)

*D*—H⋯*A*	*D*—H	H⋯*A*	*D*⋯*A*	*D*—H⋯*A*
C3—H3⋯O1^i^	0.93	2.46	3.290 (4)	149
C4—H4*B*⋯O2^i^	0.97	2.58	3.467 (5)	152
C10—H10⋯O2^ii^	0.93	2.42	3.318 (5)	162

Symmetry codes: (i) 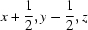; (ii) 
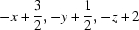
.

**Fig. 1.**
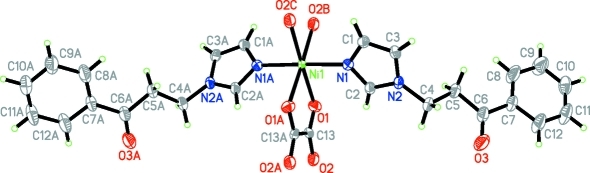
The asymmetric unit of the title compound, showing the 30% probability ellipsoids. [Symmetry codes: (A) 1-x, y, 3/2-z; (B) x, -1+y, z; (C) 1-x, -1+y, 3/2-z.]

**Fig. 2.**
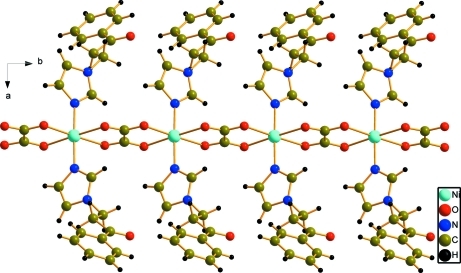
The one-dimensional structure of the title compound.

## supplementary crystallographic information

### Comment

Currently, the rational construction of new structurally defined metal-organic frameworks using a mixed-ligand strategy seems to be a marvelous success (Du *et al.*, 2005; Tao *et al.*, 2000; Ye *et al.*, 2005). In our recent research, we have initiated a synthetic approach employing multicarboxylates and 3-(1*H*-imidazol-1-yl)-1-phenylpropan-1-one (*L*) upon reactions with differnent metal ions to construct new functional frameworks. To explore this series, we synthesized the title compound, a new Ni(II) complex based on the *L* ligand.

In the title complex (Fig. 1), the Ni^II^ atom, lying on a twofold rotation axis, is six-coordinated in a distorted octahedral geometry by four O atoms from two oxalate anions in the equatorial plane and two N atoms from two monodentate *L* ligands occupying the axial positions, with the N1—Ni1—N1^i^ angle of 178.56 (17)° [symmetry code: (i) 1-*x*, *y*, 3/2-*z*]. As depicted in Fig. 2, the oxalate dianions as bridging ligands joint the Ni^II^ atoms into a one-dimensional polymeric [Ni(C_2_O_4_)]_n_ chain along [0 1 0]. The imidazole and benzene rings in the *L* ligands are not coplanar, the dihedral angel between the imidazole and benzene rings being 68.7 (2)°. Analysis of the crystal packing indicates that weak intermolecular C—H···O hydrogen bonds (Table 1) connect the chains, producing a three-dimensional supramolecular structure.

### Experimental

Ni(CH_3_CO_2_)_2_.4H_2_O (24.9 mg, 0.1 mmol), 3-(1*H*-imidazol-1-yl)-1-phenylpropan-1-one (22.2 mg, 0.1 mmol) and oxalic acid were mixed in a CH_3_CN/H_2_O solution (20 ml, v/v = 1:1) with vigorous stirring for *ca* 30 min. The resulting solution was filtered and left to stand at room temperature. Green block crystals of the title compound suitable for X-ray analysis were obtained in 65% yield by slow evaporation of the solvent over a period of one week. Analysis, calculated for C_26_H_24_N_4_NiO_6_: C 57.07, H 4.42, N 10.24%; found: C 57.13, H 4.47, N 10.32%.

### Refinement

Although all H atoms were visible in difference Fourier maps, they were finally placed in geometrically calculated positions and refined as riding atoms, with C—H = 0.93 (CH) and 0.97 (CH_2_) Å and with *U*_iso_(H) = 1.2*U*_eq_(C).

### Crystal data

**Table:**

[Ni(C_2_O_4_)(C_12_H_12_N_2_O)_2_]	*F*(000) = 1136
*M**_r_* = 547.20	*D*_x_ = 1.531 Mg m^−^^3^
Monoclinic, *C*2/*c*	Mo *K*α radiation, λ = 0.71073 Å
Hall symbol: -C 2yc	Cell parameters from 1107 reflections
*a* = 15.3065 (11) Å	θ = 2.9–21.3°
*b* = 5.6605 (4) Å	µ = 0.87 mm^−^^1^
*c* = 27.536 (2) Å	*T* = 296 K
β = 95.613 (1)°	Block, colorless
*V* = 2374.3 (3) Å^3^	0.28 × 0.22 × 0.20 mm
*Z* = 4	

### Data collection

**Table:**

Bruker APEXII CCD diffractometer	2105 independent reflections
Radiation source: fine-focus sealed tube	1676 reflections with *I* > 2σ(*I*)
graphite	*R*_int_ = 0.043
φ and ω scans	θ_max_ = 25.0°, θ_min_ = 2.7°
Absorption correction: multi-scan (*SADABS*; Sheldrick, 1996)	*h* = −16→18
*T*_min_ = 0.793, *T*_max_ = 0.845	*k* = −6→5
5794 measured reflections	*l* = −32→32

### Refinement

**Table:**

Refinement on *F*^2^	Primary atom site location: structure-invariant direct methods
Least-squares matrix: full	Secondary atom site location: difference Fourier map
*R*[*F*^2^ > 2σ(*F*^2^)] = 0.045	Hydrogen site location: inferred from neighbouring sites
*wR*(*F*^2^) = 0.096	H-atom parameters constrained
*S* = 1.02	*w* = 1/[σ^2^(*F*_o_^2^) + (0.0315*P*)^2^ + 4.5895*P*] where *P* = (*F*_o_^2^ + 2*F*_c_^2^)/3
2105 reflections	(Δ/σ)_max_ < 0.001
168 parameters	Δρ_max_ = 0.63 e Å^−^^3^
1 restraint	Δρ_min_ = −0.45 e Å^−^^3^

### Fractional atomic coordinates and isotropic or equivalent isotropic displacement parameters (Å^2^)

**Table:**

	*x*	*y*	*z*	*U*_iso_*/*U*_eq_	
Ni1	0.5000	−0.13748 (10)	0.7500	0.02887 (19)	
O1	0.46495 (16)	0.1678 (4)	0.79304 (9)	0.0464 (6)	
O2	0.46148 (17)	0.5597 (5)	0.79161 (9)	0.0528 (7)	
O3	0.8709 (3)	0.4212 (6)	0.93819 (12)	0.0912 (12)	
N1	0.62123 (18)	−0.1330 (5)	0.78683 (10)	0.0388 (7)	
N2	0.73701 (18)	−0.0047 (5)	0.83297 (10)	0.0400 (7)	
C1	0.6893 (2)	−0.2891 (6)	0.78450 (13)	0.0418 (9)	
H1	0.6865	−0.4274	0.7662	0.050*	
C2	0.6534 (2)	0.0357 (7)	0.81664 (12)	0.0421 (9)	
H2	0.6216	0.1666	0.8252	0.050*	
C3	0.7610 (2)	−0.2127 (7)	0.81268 (13)	0.0433 (9)	
H3	0.8155	−0.2863	0.8173	0.052*	
C4	0.7933 (2)	0.1504 (7)	0.86521 (13)	0.0497 (10)	
H4A	0.7668	0.3059	0.8657	0.060*	
H4B	0.8499	0.1668	0.8525	0.060*	
C5	0.8066 (3)	0.0541 (8)	0.91673 (13)	0.0591 (12)	
H5A	0.7498	0.0192	0.9279	0.071*	
H5B	0.8394	−0.0925	0.9166	0.071*	
C6	0.8547 (3)	0.2235 (9)	0.95153 (15)	0.0573 (11)	
C7	0.8814 (3)	0.1443 (9)	1.00249 (14)	0.0572 (11)	
C8	0.8604 (4)	−0.0672 (11)	1.02023 (18)	0.099 (2)	
H8	0.8268	−0.1721	1.0002	0.119*	
C9	0.8881 (5)	−0.1321 (11)	1.06808 (19)	0.115 (2)	
H9	0.8725	−0.2788	1.0797	0.138*	
C10	0.9371 (3)	0.0151 (11)	1.09733 (17)	0.0782 (15)	
H10	0.9546	−0.0283	1.1294	0.094*	
C11	0.9609 (4)	0.2238 (12)	1.08058 (18)	0.0918 (18)	
H11	0.9964	0.3247	1.1006	0.110*	
C12	0.9326 (4)	0.2895 (10)	1.03341 (16)	0.0855 (17)	
H12	0.9488	0.4367	1.0222	0.103*	
C13	0.4784 (2)	0.3633 (6)	0.77433 (12)	0.0357 (8)	

### Atomic displacement parameters (Å^2^)

**Table:**

	*U*^11^	*U*^22^	*U*^33^	*U*^12^	*U*^13^	*U*^23^
Ni1	0.0301 (3)	0.0254 (3)	0.0291 (3)	0.000	−0.0074 (2)	0.000
O1	0.0468 (15)	0.0402 (14)	0.0522 (15)	0.0059 (12)	0.0044 (12)	0.0031 (11)
O2	0.0515 (16)	0.0561 (18)	0.0480 (16)	0.0131 (13)	−0.0091 (13)	−0.0136 (13)
O3	0.125 (3)	0.075 (3)	0.066 (2)	−0.038 (2)	−0.027 (2)	0.0008 (18)
N1	0.0409 (17)	0.0349 (16)	0.0391 (16)	0.0020 (14)	−0.0034 (13)	−0.0032 (14)
N2	0.0357 (17)	0.0447 (18)	0.0372 (16)	0.0003 (14)	−0.0086 (13)	−0.0031 (15)
C1	0.042 (2)	0.040 (2)	0.042 (2)	0.0066 (17)	0.0002 (17)	−0.0078 (17)
C2	0.039 (2)	0.043 (2)	0.041 (2)	0.0053 (17)	−0.0081 (17)	−0.0050 (18)
C3	0.033 (2)	0.048 (2)	0.048 (2)	0.0055 (17)	−0.0039 (17)	0.0025 (18)
C4	0.045 (2)	0.055 (2)	0.046 (2)	−0.008 (2)	−0.0118 (18)	−0.006 (2)
C5	0.065 (3)	0.060 (3)	0.048 (2)	−0.011 (2)	−0.017 (2)	−0.002 (2)
C6	0.056 (3)	0.067 (3)	0.046 (2)	−0.008 (2)	−0.007 (2)	−0.005 (2)
C7	0.053 (2)	0.073 (3)	0.042 (2)	−0.010 (2)	−0.0081 (19)	−0.010 (2)
C8	0.133 (5)	0.095 (4)	0.060 (3)	−0.046 (4)	−0.035 (3)	0.004 (3)
C9	0.169 (6)	0.106 (5)	0.062 (3)	−0.048 (5)	−0.038 (4)	0.019 (3)
C10	0.077 (3)	0.109 (4)	0.046 (3)	0.002 (3)	−0.010 (3)	−0.008 (3)
C11	0.084 (4)	0.134 (5)	0.053 (3)	−0.036 (4)	−0.014 (3)	−0.015 (3)
C12	0.093 (4)	0.110 (4)	0.050 (3)	−0.042 (3)	−0.010 (3)	−0.009 (3)
C13	0.0311 (18)	0.0335 (19)	0.0402 (19)	0.0031 (16)	−0.0089 (15)	0.0039 (17)

### Geometric parameters (Å, °)

**Table:**

Ni1—N1	2.026 (3)	C4—H4A	0.9700
Ni1—N1^i^	2.026 (3)	C4—H4B	0.9700
Ni1—O2^ii^	2.175 (3)	C5—C6	1.497 (5)
Ni1—O2^iii^	2.175 (3)	C5—H5A	0.9700
Ni1—O1	2.191 (3)	C5—H5B	0.9700
Ni1—O1^i^	2.191 (3)	C6—C7	1.492 (6)
O1—C13	1.247 (4)	C7—C8	1.344 (7)
O2—C13	1.247 (4)	C7—C12	1.372 (6)
O3—C6	1.211 (5)	C8—C9	1.393 (7)
N1—C2	1.322 (4)	C8—H8	0.9300
N1—C1	1.372 (4)	C9—C10	1.337 (7)
N2—C2	1.334 (4)	C9—H9	0.9300
N2—C3	1.369 (4)	C10—C11	1.332 (7)
N2—C4	1.467 (4)	C10—H10	0.9300
C1—C3	1.352 (5)	C11—C12	1.380 (6)
C1—H1	0.9300	C11—H11	0.9300
C2—H2	0.9300	C12—H12	0.9300
C3—H3	0.9300	C13—C13^i^	1.550 (7)
C4—C5	1.515 (5)		
			
N1—Ni1—N1^i^	178.56 (17)	C5—C4—H4A	109.3
N1—Ni1—O2^ii^	89.51 (10)	N2—C4—H4B	109.3
N1^i^—Ni1—O2^ii^	91.63 (11)	C5—C4—H4B	109.3
N1—Ni1—O2^iii^	91.63 (11)	H4A—C4—H4B	108.0
N1^i^—Ni1—O2^iii^	89.51 (11)	C6—C5—C4	112.4 (4)
O2^ii^—Ni1—O2^iii^	75.98 (14)	C6—C5—H5A	109.1
N1—Ni1—O1	88.86 (10)	C4—C5—H5A	109.1
N1^i^—Ni1—O1	90.01 (11)	C6—C5—H5B	109.1
O2^ii^—Ni1—O1	178.36 (9)	C4—C5—H5B	109.1
O2^iii^—Ni1—O1	104.07 (9)	H5A—C5—H5B	107.8
N1—Ni1—O1^i^	90.01 (11)	O3—C6—C7	121.2 (4)
N1^i^—Ni1—O1^i^	88.86 (10)	O3—C6—C5	120.0 (4)
O2^ii^—Ni1—O1^i^	104.07 (9)	C7—C6—C5	118.8 (4)
O2^iii^—Ni1—O1^i^	178.36 (9)	C8—C7—C12	116.8 (4)
O1—Ni1—O1^i^	75.92 (13)	C8—C7—C6	123.8 (4)
C13—O1—Ni1	114.7 (2)	C12—C7—C6	119.4 (5)
C13—O2—Ni1^iv^	115.1 (2)	C7—C8—C9	121.1 (5)
C2—N1—C1	104.8 (3)	C7—C8—H8	119.4
C2—N1—Ni1	125.9 (2)	C9—C8—H8	119.4
C1—N1—Ni1	129.1 (2)	C10—C9—C8	120.4 (6)
C2—N2—C3	107.3 (3)	C10—C9—H9	119.8
C2—N2—C4	126.1 (3)	C8—C9—H9	119.8
C3—N2—C4	126.6 (3)	C11—C10—C9	120.0 (5)
C3—C1—N1	110.2 (3)	C11—C10—H10	120.0
C3—C1—H1	124.9	C9—C10—H10	120.0
N1—C1—H1	124.9	C10—C11—C12	119.6 (5)
N1—C2—N2	111.8 (3)	C10—C11—H11	120.2
N1—C2—H2	124.1	C12—C11—H11	120.2
N2—C2—H2	124.1	C7—C12—C11	122.0 (5)
C1—C3—N2	105.9 (3)	C7—C12—H12	119.0
C1—C3—H3	127.0	C11—C12—H12	119.0
N2—C3—H3	127.0	O2—C13—O1	125.8 (3)
N2—C4—C5	111.6 (3)	O2—C13—C13^i^	116.9 (2)
N2—C4—H4A	109.3	O1—C13—C13^i^	117.3 (2)
			
N1—Ni1—O1—C13	91.5 (2)	C2—N2—C4—C5	105.2 (4)
N1^i^—Ni1—O1—C13	−87.6 (2)	C3—N2—C4—C5	−77.3 (5)
O2^iii^—Ni1—O1—C13	−177.1 (2)	N2—C4—C5—C6	−173.1 (3)
O1^i^—Ni1—O1—C13	1.18 (18)	C4—C5—C6—O3	7.0 (7)
O2^ii^—Ni1—N1—C2	170.1 (3)	C4—C5—C6—C7	−173.5 (4)
O2^iii^—Ni1—N1—C2	−114.0 (3)	O3—C6—C7—C8	175.5 (5)
O1—Ni1—N1—C2	−9.9 (3)	C5—C6—C7—C8	−4.0 (7)
O1^i^—Ni1—N1—C2	66.0 (3)	O3—C6—C7—C12	−6.5 (7)
O2^ii^—Ni1—N1—C1	−5.1 (3)	C5—C6—C7—C12	173.9 (5)
O2^iii^—Ni1—N1—C1	70.8 (3)	C12—C7—C8—C9	1.3 (9)
O1—Ni1—N1—C1	174.9 (3)	C6—C7—C8—C9	179.3 (6)
O1^i^—Ni1—N1—C1	−109.2 (3)	C7—C8—C9—C10	−0.6 (11)
C2—N1—C1—C3	−0.1 (4)	C8—C9—C10—C11	−1.0 (10)
Ni1—N1—C1—C3	175.9 (2)	C9—C10—C11—C12	1.8 (9)
C1—N1—C2—N2	0.1 (4)	C8—C7—C12—C11	−0.5 (9)
Ni1—N1—C2—N2	−176.0 (2)	C6—C7—C12—C11	−178.6 (5)
C3—N2—C2—N1	−0.1 (4)	C10—C11—C12—C7	−1.1 (9)
C4—N2—C2—N1	177.8 (3)	Ni1^iv^—O2—C13—O1	177.3 (3)
N1—C1—C3—N2	0.0 (4)	Ni1^iv^—O2—C13—C13^i^	−1.6 (4)
C2—N2—C3—C1	0.1 (4)	Ni1—O1—C13—O2	178.1 (3)
C4—N2—C3—C1	−177.8 (3)	Ni1—O1—C13—C13^i^	−3.0 (4)

Symmetry codes: (i) −*x*+1, *y*, −*z*+3/2; (ii) −*x*+1, *y*−1, −*z*+3/2; (iii) *x*, *y*−1, *z*; (iv) *x*, *y*+1, *z*.

### Hydrogen-bond geometry (Å, °)

**Table:**

*D*—H···*A*	*D*—H	H···*A*	*D*···*A*	*D*—H···*A*
C3—H3···O1^v^	0.93	2.46	3.290 (4)	149
C4—H4B···O2^v^	0.97	2.58	3.467 (5)	152
C10—H10···O2^vi^	0.93	2.42	3.318 (5)	162

Symmetry codes: (v) *x*+1/2, *y*−1/2, *z*; (vi) −*x*+3/2, −*y*+1/2, −*z*+2.
